# The Week's Work

**Published:** 1910-01-22

**Authors:** 


### The Week's Work.

### AN ECHO OF THE INQUESTS.

The committee of the Battersea Antivivisection Hospital has issued a formal explanatory statement in connection with the two cases investigated by Coroner Troutbeck a fortnight ago. The details of these cases will be familiar to our readers, since they were reported in our issue of January 8. The committee's statement has been published in the *Times*, and a copy has been forwarded to us. It is scarcely convincing, since it does not explain why the resident medical officer is not put in a position to see all emergencies whenever they arise. No justification for the hospital's action is given, and as the explanatory statement stands it does not strengthen the case for the hospital, nor does it give the reader the impression that the censure passed on the institution by the coroner's jury was unnecessary or unjust. The public wants something more than an allegation that the hospital has been treated unfairly. There was no unfairness in the action taken by the coroner, nor was that action in any way different from what is customary in inquests upon hospital patients. What is wanted from the committee is an assurance that the Battersea Antivivisection Hospital will be brought into line with other general hospitals, and that it will be managed as efficiently as other general hospitals are. Unless it gives that assurance the committee cannot be surprised if the public pay more attention to the censure of the coroner's jury than to the committee's letter in the *Times*.

### PUTTING THEIR HOUSE IN ORDER.

The fact is that the authorities at the Battersea Hospital have been very lax, and far too much occupied with controversy. These inquests have shown clearly that their house is not in order, and that their hospital cannot demand to be looked upon as a well-conducted institution in which the public can repose confidence. It is not a question of men; it is one of management and method, and we want an assurance from the Board that the methods of the institution will be modern and efficient. We are not now concerned with the controversial side of the hospital, though we must confess that it is a relief to hear, upon oath, that antiseptics are used, and that Listerian technique is not wholly ignored at the Battersea Anti-Vivisection Hospital. But the administrative side of the hospital has nothing to do with anti-vivisection. No anti-vivisection board can object to keeping proper out-patient books, which can be produced in evidence, and should be so produced when, as in one of the inquest cases, there is a question of a possible conflict of evidence; to having proper cards of instruction printed for the use of out-patients; and to provide a resident medical officer and an assistant resident medical officer to ensure that properly qualified assistance is always at hand in case of an emergency. That being the case, we are glad to publish the following official letter from the Hospital Secretary, which shows that the Board has at last realised its responsibilities in the matter of hospital management:

National Anti-Vivisection Hospital,
The Battersea General Hospital,
Battersea Park, London, S.W.,
January 12.

At a meeting of the Board of Management held on Wednesday, January 12, the Board appointed both a house surgeon and a house physician. This step has been contemplated for some time owing to the growth of the hospital's work in the district. A duly qualified medical officer will consequently in future always be in attendance at the hospital to receive patients.

(Signed) George W. F. Robbins, Secretary.

This is a step that we have already advocated on more than one occasion, and if these posts are efficiently filled there ought to be no risk in future of dragging the hospital through a coroner's court. The Board should see that it gets a thoroughly competent and efficient resident staff. That, in the circumstances, and owing to the peculiar conditions that it asks its officials to subscribe to, will no doubt be a matter of some difficulty, but at any rate the first step in the right direction has been taken by arriving at the above resolution.

### PAY YOUR WAY.

It is interesting to observe that the more the financial position of the London hospitals improves as a whole, the more pessimistic some critics become. Fifteen years ago it would have been true and desirable to point out that several of the hospitals were outrunning the constable and that their managers ought to cry a halt. At that time there was a deficiency of £100,000 every year at least, by which sum the voluntary contributions to hospitals in London were short of the expenditure, when all items of revenue were included. The King's Fund was instituted in 1897 to adjust the balance and to provide this additional voluntary revenue. In fact, this fund gave the hospitals £145,000 in 1909. We have published the figures for all voluntary hospitals more than once recently, which show that financially the voluntary hospitals of London and all voluntary hospitals in the United Kingdom were never in a sounder financial position than they are to-day. Some stir has been made because a will recently proved contains a codicil revoking a bequest of £500 to St. Bartholomew's Hospital on the ground that this institution is exceeding its income. We are glad to note this codicil, because it emphasises a view, frequently maintained in these columns, that no voluntary hospital is entitled to be treated with generous confidence by the public unless it is managed upon business principles. The management of St. Bartholomew's Hospital during the last ten years has not been good business, and the effect of this mismanagement must be felt pretty keenly by the new treasurer, Lord Sandhurst, who has thus been placed in a very awkward position.

### AN EXPLODED IDEA.

The old idea that a voluntary hospital must show a deficit in order to secure liberal contributions from the public does not apply to the most generous and knowledgeable supporters of our voluntary hospitals to-day. They believe rightly that every hospital committee should commence the year by considering detailed estimates of the income and expenditure for such year, and upon these figures every committee should resolve, as many modern hospitals do, to base its expenditure. As we show in another column, the public as a whole desires a change in the present system of admission of in-patients to our voluntary hospitals. The majority of those who seek in-patient relief would prefer to have an opportunity to pay a portion at any rate of the cost entailed by their treatment. We advocate a reform in this direction, and would urge hospital committees, when each year's budget comes before them, to apportion a certain number of beds to pay-patients and so to provide that each year's revenue shall cover each year's expenditure. If the action of the testator in regard to St. Bartholomew's leads to a reconsideration of the present methods of hospital finance, it will have done a great public service. We hope it may, but meanwhile it is desirable in our view that a large proportion of the legacies received should be devoted to current expenditure. Any hospital which has invested funds amounting to a sum equal to five years' expenditure has an ample reserve, for the duty of voluntary hospital managers is to supply the needs of the sick of to-day, leaving the future to take care of itself. Certain abuses to which endowed institutions are liable have been condemned by this testator, and, if the voluntary hospitals are to endure, as we believe they must, it will be because they continue to supply efficiently the needs of the people who require hospital aid.

### THE DRUG MARKET.

A very heavy supply of drugs was offered for sale at the recent public auction, held in Mincing Lane, and while a very fair proportion was sold, the changes in value were remarkably few. Gum asafœtida fetched extremely high prices, and Buchu leaves, not of the finest quality, realised more than twice the price that was paid a few months ago. Cape aloes was slightly dearer. In the case of most of the other drugs prices were well maintained, and on the whole there was a fairly good all-round demand. Apart from the transactions effected at the auctions, business has been quiet, although there has been a fair amount of inquiry which seems to promise better business in the near future. Crude carbolic acid is slightly dearer, and it is not unlikely that the prices of finer qualities may be advanced before long. Opium is in very small request at the moment, as buyers are expecting the value to recede, but it is difficult to say whether this expectation will be realised; it would be wise, however, to refrain from buying in any quantity at the moment. Citric acid is tending dearer.

